# Familial bias and auditory feedback regulation of vocal babbling patterns during early song development

**DOI:** 10.1038/srep30323

**Published:** 2016-07-22

**Authors:** Daisuke Sato, Chihiro Mori, Azusa Sawai, Kazuhiro Wada

**Affiliations:** 1 Graduate School of Life Science, Hokkaido University, Sapporo, Hokkaido, Japan; 2 Department of Biological Sciences, Hokkaido University, Sapporo, Hokkaido, Japan; 3 Faculty of Science, Hokkaido University, Sapporo, Hokkaido, Japan

## Abstract

Learned vocalizations are a crucial acoustic biosignal conveying individual traits in many species. Songbirds learn song patterns by listening to a tutor song and performing vocal practice during a sensitive developmental period. However, when and how individual differences in song patterns develop remain unknown. Here, we report that individual differences in vocal output exist even at the earliest song development stage, called subsong. Experiments involving the manipulation of both breeding pairs and song tutoring conditions revealed that the parental pair combination contributes to generating familial differences in syllable duration and variability in the subsong of offspring. Furthermore, after deafening, juveniles immediately changed their subsong by shortening the syllable durations but maintained the individual variability of their subsong temporal patterns, suggesting both auditory-sensitive modification and independent intrinsic regulation of vocal output. These results indicate that the temporal patterns of subsong are not merely disordered vocalization but are regulated by familial bias with sensitivity to auditory feedback, thus generating individual variability at the initiation of vocal development.

Birdsong is a learned behavior that is used as an acoustic biosignal to distinguish between individuals for mating and territorial defense^1^. Among songbirds, the zebra finch acquires its song patterns through two phases: the sensory-learning and sensorimotor-learning phases^2^,^3^. During the sensory-learning phase, juveniles acquire the sensory memories of songs by listening to the songs of adult birds as a song template to be imitated. The sensorimotor-learning phase starts with the generation of soft and highly variable syllables, in a song-developing state called subsong. Subsongs are generated mainly as vocal output from the neural activity of song nuclei in the basal ganglia–thalamocortical loops in the song system^4^,^5^. Thereafter, birds start producing what is called plastic song, which is the gradual inclusion of recognizable yet variable syllables. At the end of the learning process, the song is crystallized with acoustically and sequentially stable syllable patterns. During this learning process, auditory feedback is crucial for monitoring vocal outputs to match them with memorized tutor song patterns^6^.

However, in the zebra finch (*Taeniopygia guttata*), even when juveniles listen to the same tutor song during the sensory-learning phase, they generate individually unique song patterns and maintain species-specificity in crystallized song at maturity^7^. Furthermore, during the sensorimotor-learning phase, zebra finch juveniles use multiple learning strategies to develop their song patterns^8^. Given the existing evidence suggesting the developmental origin of individual differences in learned vocalization, we investigated when and how zebra finches initiate the generation of individual variation in song patterns and how auditory feedback contributes to the regulation of vocal output during the early critical period for vocal learning. Here, we show that individual variation in song temporal patterns exists at the initiation of song development, *i.e.*, in the early subsong stage. Furthermore, experiments combining the manipulation of breeding pairs and tutoring conditions revealed that the temporal pattern of juvenile subsongs differed among breeding families but was scarcely influenced by the tutored song type. Although auditory feedback contributed to the regulation of syllable duration in subsong, the individual differences in subsong temporal patterns persisted after deafening.

## Results

### Consistency and individual differences in the temporal patterns of zebra finch subsong

**Figure 1:**
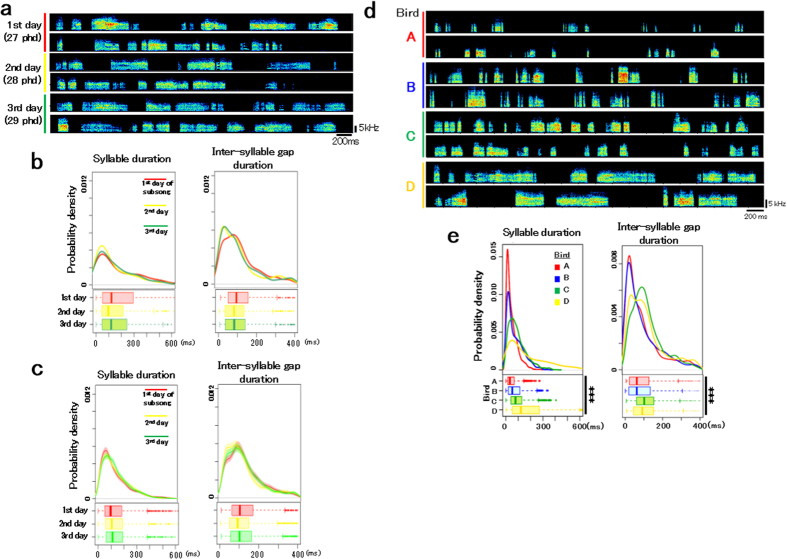
Consistency and individual differences in the subsong temporal patterns of zebra finches. (**a**) An example of subsong during three consecutive days after the onset of subsong in a zebra finch male. (**b**) Temporal patterns of the same bird (**a**) on each of the three days. Distribution of probability densities (upper box) and boxplot (lower box) of the syllable and inter-syllable gap durations. (**c**) Average temporal pattern distributions of subsong on three consecutive days after the onset of subsong (n = 4 birds). Shaded areas in the distribution of probability density indicate 95% confidence interval. (**d**) Examples of individual differences in the subsong pattern on the first day of subsong singing in four juvenile zebra finch males (n = 4 birds each from four different families). (**e**) Distribution of the probability densities of the syllable and inter-syllable gap durations of subsongs in the four juveniles shown in panel (d). (1,200 syllables/bird from initial subsong production; ***p < 2.2e-16 for the duration of syllables and inter-syllable gaps, Kruskal-Wallis test). In the box plots shown in (**b**,**c**,**e**), the edges of boxes indicate the upper- and lower-quartile of each group, respectively. The whiskers indicate the most extreme data point that is no more than 1.5 times the inter-quartile range from the box. Dots indicate the outliers.

To evaluate the beginning of the emergence of individual differences in song patterns, we continuously recorded sounds in breeding cages before the male juveniles started to sing. Subsong is observed as vocal babbling lacking stereotypy in both the acoustic structure and sequence of syllables^4^. Therefore, we focused on four temporal parameters of song structure: both the duration and its variance of syllables and inter-syllable gaps, which are the fundamental parameters forming the temporal features of the song pattern. To measure the variability in the duration of syllables and inter-syllable gaps, we used the value of the interquartile range (IQR) normalized by the median (as the IQR/med). We first examined the developmental changes in subsong patterns by comparing the temporal parameters of individual subsongs for three consecutive days after initiation of subsong. All the temporal parameters of the subsongs were consistent across the first three days of subsong [using 334–400 (average 383.5) syllables/day for each bird (n = 4), Fig. 1a–c]. Individual variability in the temporal patterns of subsong was explicitly observed among juvenile zebra finch males (n = 4 birds from four different families, Fig. 1d). An individual difference was found in the duration of both syllables and inter-syllable gaps, even at the early onset of subsong production (1,200 syllables/bird from initial subsong production; ***p < 2.2e-16 for the duration of syllables and inter-syllable gaps, Kruskal-Wallis test) (Fig. 1d,e). These results encouraged us to further investigate the causal factors affecting the generation of individual differences in song structures at the time of subsong initiation.

### Familial bias due to parental pair combination on the temporal structures of subsong in offspring

**Figure 2:**
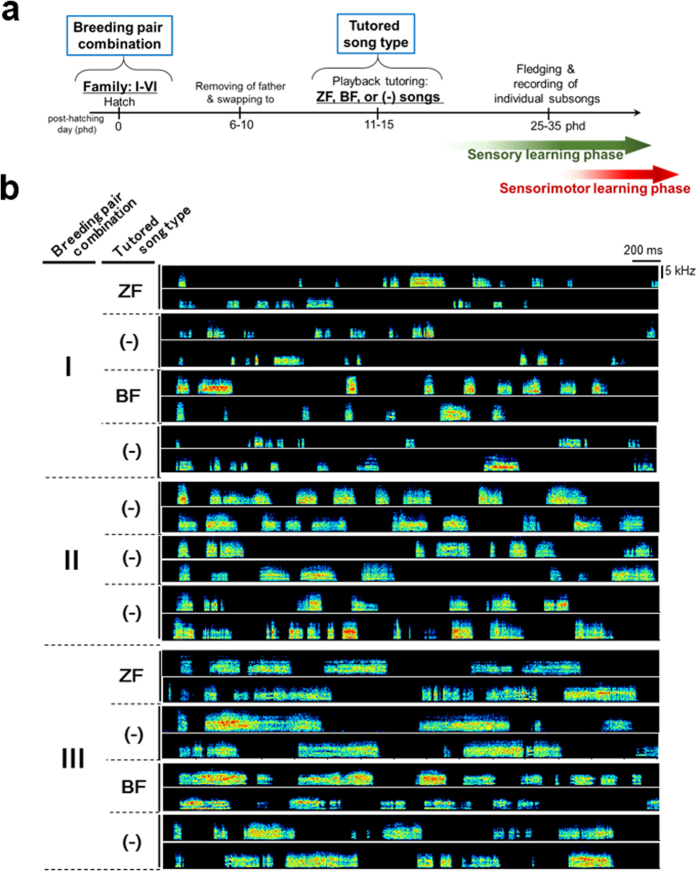
Subsongs of juvenile zebra finches reared with tutoring manipulation. (**a**) Outline of the experiment with tutoring manipulation. (**b**) Examples of subsongs from juvenile zebra finches reared under the experimental combinations described above from breeding families I, II, and III. ZF, BF, and (−) mean playback conditions with zebra finch songs, Bengalese finch songs, and no song tutoring, respectively.

To elucidate the potential factors affecting the regulation of subsong structure, we experimentally manipulated two factors in the rearing of zebra finch chicks: breeding pair and tutored song type (Fig. 2a). We prepared six breeding pairs to obtain sufficient numbers of male juveniles. Chicks were separated from their fathers by between 6 and 10 post-hatching day (phd) to avoid juveniles listening to their father’s songs as tutor songs, and we then either moved the chicks into other nests maintained by genetically unrelated females or kept them in the same nest with their biological mothers to adjust the clutch size in a nest. One of the following three tutoring conditions was used for juveniles: playback of a genetically unrelated zebra finch (ZF) song, playback of a hetero-specific Bengalese finch (BF) song, or no playback. The song of the Bengalese finch was chosen for its acoustic features and the sequence of syllables, which were rarely observed in the songs of zebra finches (Supplementary Fig. S1). We hypothesized that tutor song types might be a crucial factor regulating the subsong patterns of juveniles and that tutoring birds with the Bengalese finch song would affect the temporal patterning of the subsongs in different ways than would tutoring with the zebra finch song. Tutoring by song playback was initiated between 11 and 15 phd, before the fledgling stage and before the onset of song learning and song tutor selection in zebra finches^9^ (Fig. 2a). Finally, we obtained a set of subsongs from 25 male juveniles (mean phd ± SD = 33.3 ± 5.0) that were reared under different combinations of breeding pairs and tutored song types (Fig. 2b, Supplementary Table S1).

**Figure 3:**
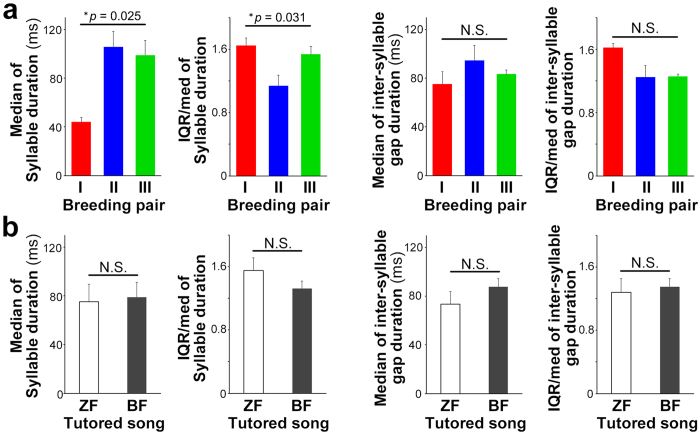
Familial breeding pair bias in temporal patterns of subsongs in offspring. (**a**) Differences in the median and IQR/median of the subsong syllable duration, but not the inter-syllable gap duration of non-tutored juveniles among breeding pairs (n = 4, 6, 4 from breeding pair I, II, and II, respectively) (*p = 0.025 and 0.031 for the median and IQR/median of the subsong syllable duration, respectively. *p* = 0.632 and 0.185 for the median and IQR/median of the inter-syllable gap duration, respectively. One-factor ANOVA). Error bars: s.e.m. (**b**) No significant differences in the median and IQR/median of the duration of subsong syllables and inter-syllable gaps between ZF and BF song tutoring juveniles (n = 5 and 6, respectively) (*p* = 0.845 and 0.235, the median and IQR/median of the subsong syllable duration, respectively. *p* = 0.272 and 0.717 for the median and IQR/median of the inter-syllable gap duration, respectively. Student’s t-test). Error bars: s.e.m.

We tested the effects of parental breeding pair on the temporal features of subsongs in the juveniles that did not experience tutored songs, to minimize any potential effects of song tutoring. We then compared the temporal features of subsongs of offspring from three different breeding pairs (n = 4, 6, and 4 from Breeding pair I, II, and III, respectively; Supplementary Table S1). Significant differences among breeding pairs were observed in both the median and IQR/med of the syllable duration (*p = 0.025 and 0.031 for the median and IQR/med, respectively; one-factor ANOVA) (Fig. 3a). However, we found no significant effects of breeding pairs on the temporal features of the inter-syllable gaps in the juvenile subsongs (Fig. 3a).

We then examined the potential effects of tutored song type on the generation of subsong temporal patterns by comparing ZF and BF song tutoring. In contrast to the results of parental breeding pair manipulation, there was no significant difference in any temporal features of song when comparing the subsongs of birds tutored with different song types (Fig. 3b). On the basis of the results indicating a potential contribution of familial bias in the generation of subsong patterns, we then assessed to which degree did auditory feedback contribute to the temporal patterning of subsong because it is well known that monitoring of own vocal outputs by auditory feedback is crucial for development and regulation of syllable acoustic and sequential patterns^10^,^11^,^12^. Furthermore, an auditory deprivation experiment would be able to more clearly elucidate any possible contribution of innate factors for regulation of the subsong pattern.

### Auditory feedback contribution to the regulation of subsong structures

**Figure 4:**
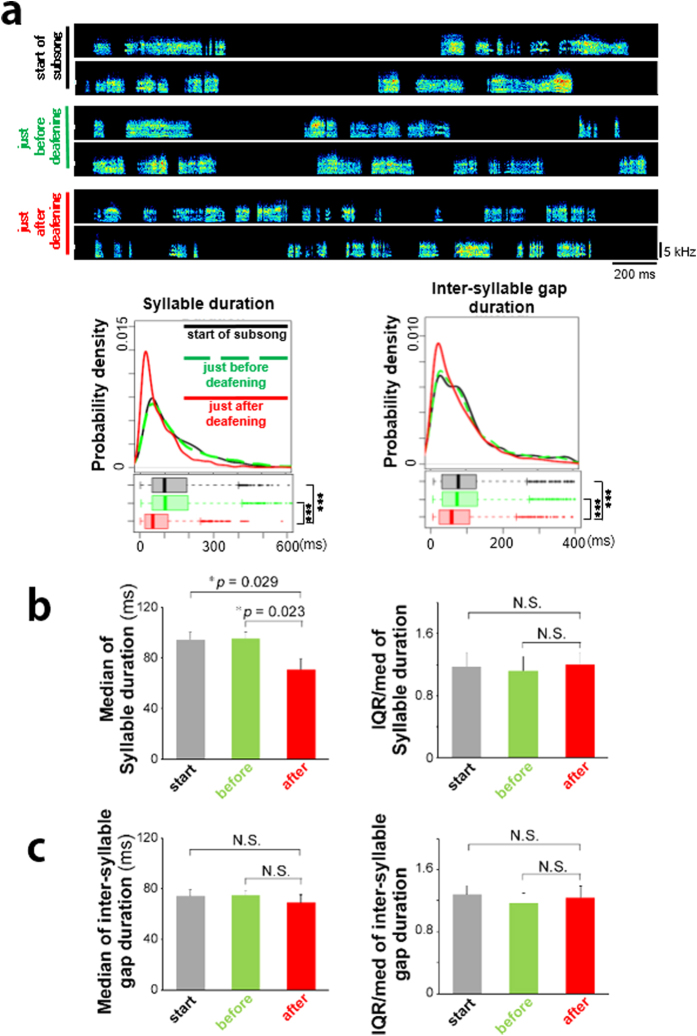
Effect of deafening on the temporal patterns of subsong and persistent individual differences of subsong patterns after deafening. (**a**: upper) An example of subsong of a juvenile zebra finch at 33 phd, just before deafening at 34 phd, and just after deafening at 36 phd. (**a**: bottom) Temporal patterns of the same bird (a) on each day. Distributions of the probability density (upper box) and boxplot (lower box) (***p < 1.0e-5: Wilcoxon signed-rank test). **(b)** Median and IQR/median of the syllable duration in subsongs (n = 5 birds) during the start of subsong (black), before deafening (green), and after deafening (red). (*p = 0.029 and 0.023: paired t-test after Holm’s correction). Error bars: s.e.m. **(c)** Median and IQR/median of the inter-syllable gap duration in subsongs (n = 5 birds) during the start of subsong (black), before deafening (green), and after deafening (red). Error bars: s.e.m.

To examine whether subsong pattern is regulated by auditory feedback, juvenile zebra finches were deafened after observing subsong initiation and the temporal structures of the subsong before and after deafening were compared (Fig. 4a). The deafening procedure and song recording were performed within three days after subsong initiation. Within the period, the temporal parameters in subsongs were consistently stable (Fig. 1a–c). Although there were no significant differences in the generation of syllables and inter-syllable gaps between the first day of the subsong onset and just before the deafening operation, we found that the syllable duration of the subsong was affected differently after deafening (Fig. 4b). The effects of deafening on subsong were immediately and consistently observed as a decrease in syllable duration in all deafened birds (n = 5 birds, 1,200 syllables/time point, *p = 0.029 and 0.023 for “start vs after” and “before vs after” of the median, respectively; paired t-test after Holm’s correction) (Fig. 4a,b). In contrast, the temporal features of the inter-syllable gap showed no significant changes after deafening (Fig. 4c). No distinct changing, such as decreasing of syllable duration, was observed in sham-deafened birds (Supplementary Fig. S2).

### Persistent individual variability of subsong temporal patterns after deafening

**Figure 5:**
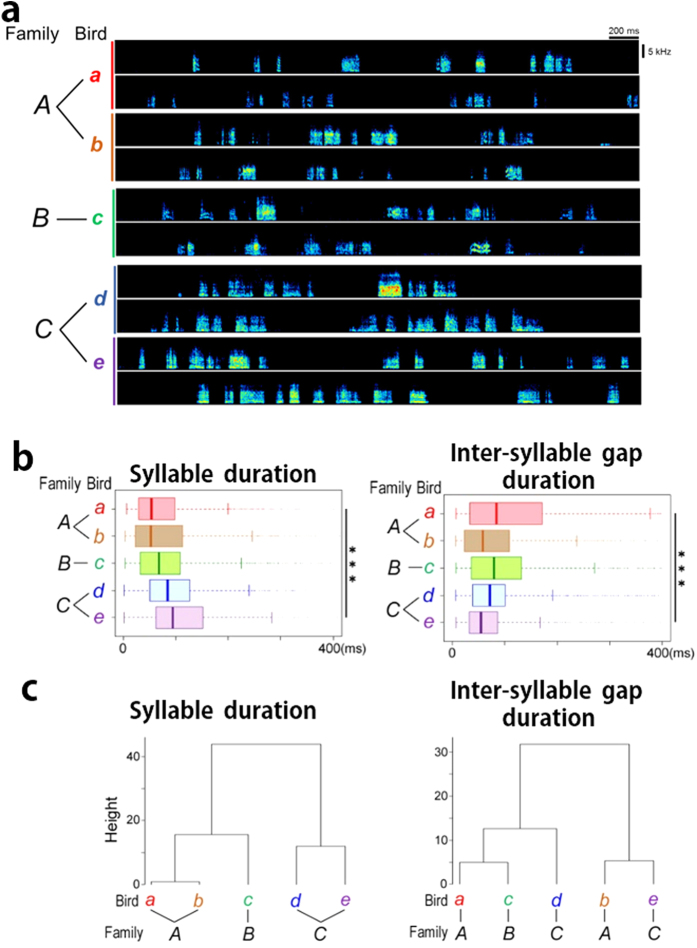
Persistent individual variability of subsong temporal patterns after deafening. (**a**) Examples of subsong of the deafened juvenile zebra finches (n = 5 from three different breeding pairs). Birds “*a* and *b*” and “*d* and e” were siblings from two different breeding families *A* and *C*, respectively. (**b**) Temporal patterns of subsongs of the five birds after deafening shown in (**a**). Distributions of the probability density (upper box) and boxplot (lower box) (***p < 2.2e-16, Kruskal-Wallis test). (**c**) Hierarchical Cluster analyses calculating the Euclidean distance of the value of median and IQR/med of the duration of syllable and inter-syllable gap of subsongs of the deafened juveniles.

Although deafening manipulation clearly indicated that auditory feedback contributed to the regulation of subsong syllable duration, individual variability of the subsong was still explicitly observed among deafened juveniles (Fig. 5a). The duration of both syllables and the inter-syllable gaps of subsong after deafening showed significant differences among individuals (n = 5, ***p < 2.2e–16, Kruskal-Wallis test) (Fig. 5b). In addition, even in the small sample that included two pairs of male juveniles from two different breeding families, hierarchical cluster analyses revealed that the two pairs of deafened juveniles from different families were separated in the different clades of the dendrogram of the syllable duration of subsong after deafening (Fig. 5c). When we compared the before and after deafening dendrograms of the durations of syllable and inter-syllable gap, the condition of auditory deprivation showed a clearer familial bias in subsong syllable durations than the condition before deafening (Fig. 5c and Supplemental Fig. S3). This suggests a potential persistent bias on the familial regulation of the temporal pattern of syllable duration without auditory feedback.

## Discussion

This is the first study to characterize individual differences in vocal babbling, also known as subsong, in songbirds. Experiments combining the manipulation of breeding pairs and tutoring conditions showed that the breeding pair combination generates a familial bias on the regulation of the syllable duration and variability in the subsong of offspring. In addition, although we found that auditory feedback contributed to the regulation of the syllable duration of subsong from the early vocal-learning period, the individual variability of subsong temporal patterns was persistently generated with a familial bias after deafening.

Previous studies have reported a large instability in syllable acoustic and sequential features in subsong^4^,^13^. However, whether the variability in subsong possesses any characteristic traits or whether it is produced in a disorderly and random manner has yet to be examined. In this study, by measuring both the duration and variability in the syllable and inter-syllable gap in subsong, we demonstrated that subsong output was not merely disordered vocalization but was regulated by individually different variability in the duration of syllables. Experimental manipulation of breeding pair and tutoring revealed that the breeding parental combination produces a familial bias in the regulation of syllable duration and variability, suggesting the possibility of a genetic contribution to the generation of individual variability in subsong patterns. This hypothesis was supported by our deafening experiment. Although the effect of deafening on subsong was immediately and consistently observed as a decrease in syllable duration, it also revealed a persistent individual variability with a trend of familial bias in subsong patterns after auditory deprivation. These results indicate the possibility that the individual variability of subsongs was basically regulated by a genetic contribution and further modified by auditory feedback of the bird’s own vocalization. To further examine the idea of such a genetic bias, it might be valuable to compare the similarity in subsong temporal patterns among biological fathers, their offspring, and genetically unrelated males. We noticed that our experimental design in this study could not rule out other possibilities, such as the potential effects of the quality of the parental care on the subsong structure of offspring. The early parental care could contribute to differing nutritional and stress conditions of nestlings between breeding pair combinations. The time of song nuclei development in the song system coincides with the early phd for receiving the “nutritional stress” from biological parents^14^. In addition, several studies have shown that offspring development is affected by maternal effects, such as hormones in the egg yolk^15^,^16^. Therefore, the different familial bias on the generation of subsong pattern may result from a complex combination of early developmental nutritional stress and maternal effects. Further experiments are needed to evaluate the epigenetic contributions to the generation of the familial bias in subsong patterns.

Subsong is generated as vocal output from the neural activity of the basal ganglia–thalamocortical loop in the song system^4^,^13^. The neural circuits are necessary for song learning^17^,^18^,^19^ and are involved in the real-time control of song production^20^,^21^ and modification^11^ using auditory feedback^22^. The result of the deafening operation at the subsong stage provided direct evidence for the contribution of auditory feedback on the vocal output of the basal ganglia–thalamocortical loop, shown as an immediate shift toward shortened syllable duration in subsongs. The auditory-sensitive modification of vocal output might be regulated at Area X in the basal ganglia–thalamocortical loops through dopaminergic neurons in the substantia nigra pars compacta (SNc) and ventral tegmental area (VTA), which show responses to the bird’s own song and are considered to be a sender of a time-dependent reward signal^13^,^23^,^24^. It still remains unclear how auditory information contributes to modulation of the neural activity of the basal ganglia–thalamocortical loop. The persistent generation of individual variability in subsong patterns was observed in deafened juveniles. This result also suggests the existence of an auditory-independent intrinsic regulation of vocal output by the basal ganglia–thalamocortical loop. Our research group has reported individual variation in the expression of androgen receptor in the song nucleus Area X of the basal ganglia^25^. Therefore, the idea of a genetic contribution to the generation of individual variability in the temporal structures of subsong might coincide with the results showing the individual variations in a set of gene expression levels of song nuclei in the basal ganglia–thalamocortical loops.

The behavioral significance of the individual variability in the generation of subsong patterns during song learning remains to be investigated. Both the individual variability and familial bias in subsong were observed to influence syllable duration. Thus, for instance, a juvenile with a bias toward generating long-syllable duration in subsong might learn longer syllables in tutor songs more easily and quickly than other juveniles without that bias. If so, this learning preference bias should affect the juvenile song pattern learning strategy and cultural evolution of the song. Further studies are needed to examine whether the individual differences in the temporal regulation of subsongs may, as an early learning bias, directly affect the development of individual uniqueness in song development and learned song patterns.

## Methods

### Animals

For the experiment assessing the consistency of subsong temporal patterns, we used four zebra finch juveniles that sang more than 330 syllables/day in the first three days after start of the subsong stage. To examine the emergence of individual differences in subsong patterns, four zebra finch juveniles were used from four different breeding pairs. Adult male and female zebra finches used for breeding pairs were obtained from a local breeder. The photoperiod was maintained at a 13 h:11 h light:dark cycle, and food and water were provided *ad libitum*. The sex of birds was determined by polymerase chain reaction within 5 phd, as described in a previous study^26^. For experiments combined with breeding pair and tutoring manipulation, we prepared six breeding pairs to obtain sufficient numbers of male zebra finch juveniles. Between 6 and 10 phd, before juvenile zebra finches could start memorizing a tutor song^9^, juveniles were separated from their fathers and were randomly moved into a nest with an adult female, keeping a 3–4 sibling clutch size so that the juveniles received similar care. At this time, we observed no distinct abnormalities, such as age-unmatched body weight or size, in the juveniles. At 11 to 15 phd, song tutoring was set with one of the three conditions as a cross-tutoring experiment: playback of a zebra finch song, playback of a Bengalese finch song, or no playback (see Supplementary Fig. S1). Tutor songs were played five times in the morning and five times in the afternoon at 55–75 decibels from a speaker (SRS-M30, SONY) controlled by Sound Analysis Pro (v1.04)^27^. All experiments were conducted under the guidelines and approval of the Committee on Animal Experiments of Hokkaido University based on the national regulations for animal welfare in Japan (Law for the Humane Treatment and Management of Animals; after a partial amendment No. 105, 2011).

### Song recording and analyses

Birds were individually housed in a sound-attenuation box. Songs were automatically recorded and saved using Sound Analysis Pro software. Low- and high-frequency background noises in song files (<0.5 kHz and >15.8 kHz, respectively) were filtered from the recordings. Calls were eliminated from the dataset. Sounds separated by <7 ms of silence were used as a single syllable, and sounds with <7 ms duration were eliminated as noise. Syllables were segmented using the Avisoft Saslab software (Avisoft Bioacoustics), and subsequently, the separated syllables were manually double-checked for precision in syllable separation. A total of 1,200 syllables and associated inter-syllable gaps from the earliest subsongs were used for the analyses of temporal structures of the subsong. The distributions of probability density were derived for syllable and syllable gap durations with the statistical software R ver. 3.2.2^28^.

For statistical analyses, F tests were first performed to confirm the homoscedasticity of data sets. Because of a non-normal distribution of the syllable and inter-syllable durations in subsong, Kruskal-Wallis test and Wilcoxon single-rank test were performed for the comparison of individual differences of the durations in duration. The datasets of the median and IQR/med of syllable and inter-syllables durations confirmed the homoscedasticity. Therefore, we performed ANOVA and t-test for the analyses of the breeding pair and tutor song effects.

### Deafening

The deafening operation was performed as described in a previous study^29^. Five birds from three different breeding pairs were deafened by cochlear extirpation. The deafening procedure and song recording were performed within three days after subsong initiation. The birds were anesthetized with pentobarbital (6.48 mg/mL; 60 μL/10 g of body weight) by intra-peritoneal injection. After fixing of each bird’s head in a custom-made stereotaxic apparatus with ear bars, a small window was made through the neck muscle and the skull near the end of the elastic extension of the hyoid bone. The cochlea was pulled out with a fine hooked wire. The removed cochleae were observed by visual inspection under a dissecting microscope. After bilateral cochlear removal, a recovery period staying on a heat pad was set for 2–3 h, and then the birds were placed back in the sound-attenuation box to record their songs. To clarify the potential effects of the surgery procedures, such as physical constrain, anesthesia hangover, and invasive cranial surgery, on generation of subsong temporal patterns, sham-operations were performed for two birds. Sham-operations were followed as procedures described above until the step involving the opening of a small window through the skull near the end of the elastic extension of the hyoid bone. No distinct effects were observed after sham-operations as shown in the Supplementary Fig. S2.

## Additional Information

**How to cite this article**: Sato, D. *et al*. Familial bias and auditory feedback regulation of vocal babbling patterns during early song development. *Sci. Rep.* **6**, 30323; doi: 10.1038/srep30323 (2016).

## Supplementary Material

Supplementary Information
