# Supplementary material for: Familial bias and auditory feedback regulation of vocal babbling patterns during early song development
Source: Sci Rep. 2016 Jul 22;6:30323. doi: 10.1038/srep30323 (PMC4957237; doi:10.1038/srep30323)
Supplement: Supplementary Information [file srep30323-s1.pdf]

Supplementary information

Familial bias and auditory feedback regulation of vocal babbling pattern during early song development

Daisuke Sato[1], Chihiro Mori[1], Azusa Sawai[1] & Kazuhiro Wada[1, 2, 3#]

[1]Graduate School of Life Science, [2]Department of Biological Sciences, and [3]Faculty of Science, Hokkaido University, Sapporo, Hokkaido, Japan

Parental breeding pair	Tutored song type		
	(-)	ZF	BF
I	4	1	1
II	6		
III	4	1	1
IV		1	2
V		1	1
VI		1	1

Supplementary table S1: The number of juvenile zebra finch males used for the cross-fostering experiment combined with tutoring manipulation

(-), ZF, and BF indicate song playback conditions with no song tutoring, zebra finch songs, and Bengalese finch songs, respectively.

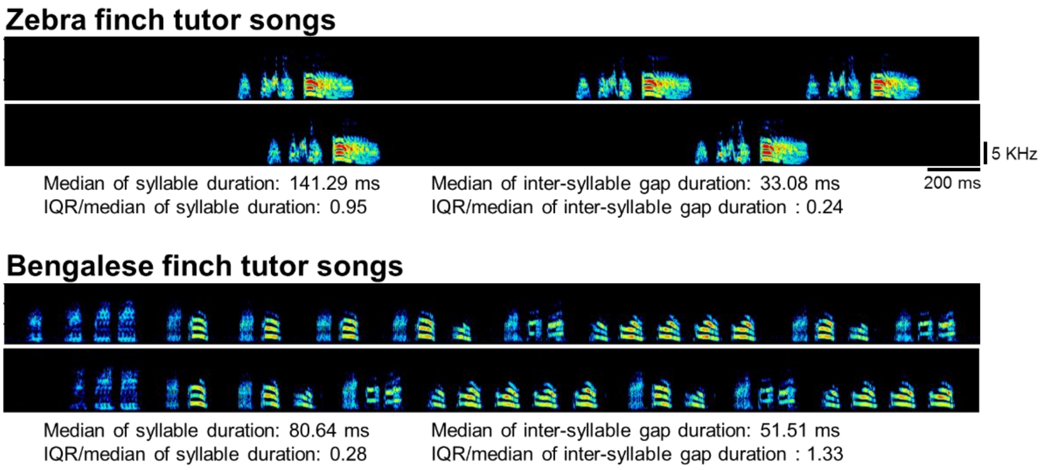

Supplementary Fig. S1: Examples of sound spectrograms of the playback songs of zebra finches and Bengalese finches

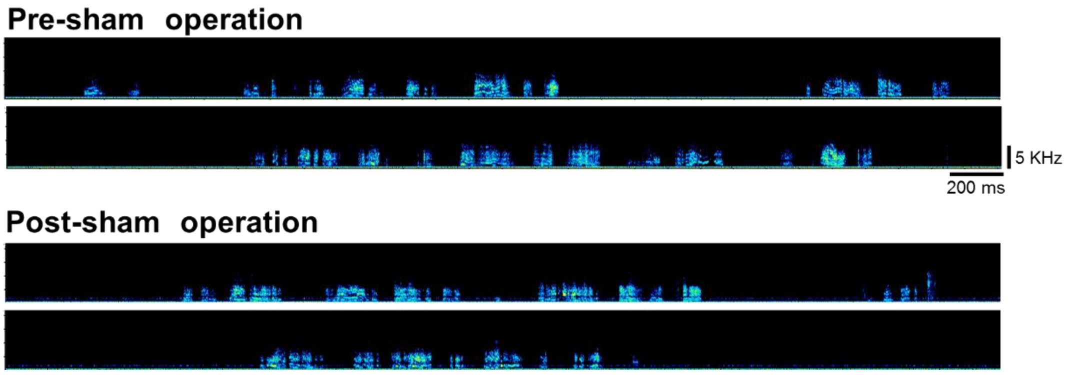

Supplementary Fig. S2: No distinct change of subsong patterns was observed after Sham-operation in sham-deafened birds.

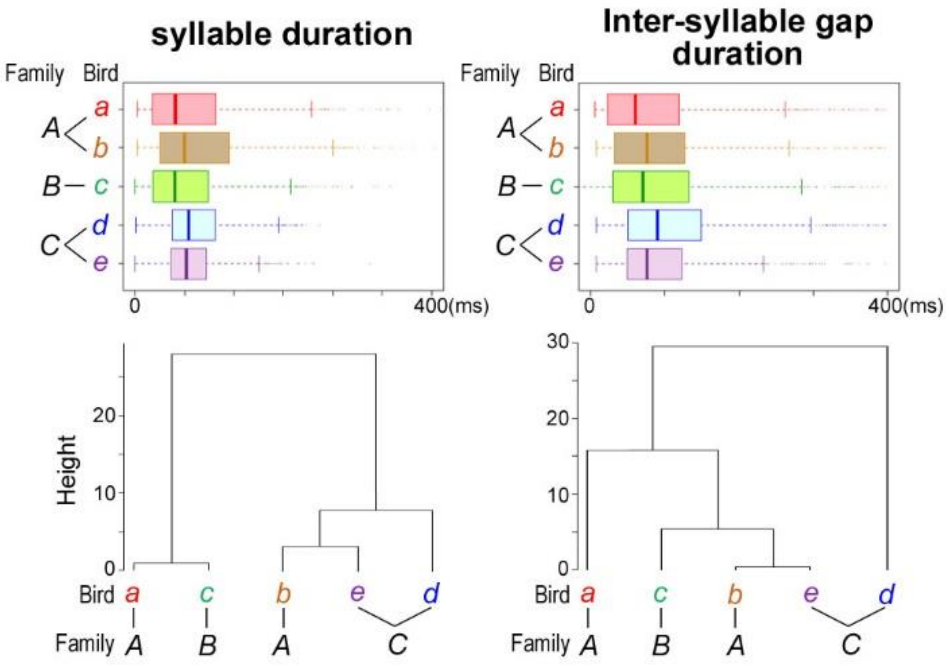

Supplementary Fig. S3: Hierarchical Cluster analyses calculating the Euclidean distance of the value of median and IQR/med of the duration of syllable and inter-syllable gap of subsongs before deafening of the same juveniles shown in Figure 5.
